# Evaluation of the biofilm-forming ability and molecular characterization of dairy *Bacillus* spp. isolates

**DOI:** 10.3389/fcimb.2023.1229460

**Published:** 2023-07-31

**Authors:** Angela Maria Catania, Pierluigi Di Ciccio, Ilario Ferrocino, Tiziana Civera, Francesca Tiziana Cannizzo, Alessandra Dalmasso

**Affiliations:** ^1^ Department of Veterinary Sciences, University of Turin, Largo P. Braccini 2, Grugliasco, Turin, Italy; ^2^ Department of Agricultural, Forest and Food Sciences, University of Turin, Largo P. Braccini 2, Grugliasco, Turin, Italy

**Keywords:** biofilm, *Bacillus*, polystyrene, stainless-steel, SEM, whole genome sequencing

## Abstract

Food processing lines represents a suitable environment for bacterial biofilm formation. One of the most common biofilm-forming genera in dairy processing plants is *Bacillus*, which includes species that may have a negative impact on safety and/or quality of dairy products. In the current study, we evaluated the biofilm forming ability and molecular characteristics of dairy *Bacillus* spp. isolates (*B. cereus* and *B. subtilis*). Reference strains (*B. cereus* ATCC 14579 and *B. subtilis* NCTC 3610) were also included in the experiment. All isolates were screened by micro-titer plate (96 wells) to assess their ability to form biofilm. Then, they were tested on two common food contact surfaces (polystyrene and stainless steel) by using 6-well plates and AISI 316 stainless steel coupons. Biofilm formation, expressed as biofilm production index (BPI), was higher on polystyrene than stainless steel (except for *B. cereus* ATCC 14579). These observations were further confirmed by scanning electron microscopy, which allowed the microscopy observation of biofilm structure. Moreover, a possible correlation among total viable cell counts (CFU) and BPI was examined, as well as a connection among biofilm formation and bacterial cell hydrophobicity. Finally, whole genome sequencing was performed highlighting a genetic similarity among the strains belonging to the same species. The presence of selected genes involved in biofilm formation was also examined showing that strains with a greater presence of these genes were able to produce more biofilm in the tested materials. Additionally, for *B. cereus* strains enterotoxin genes were detected.

## 1 Introduction

Many microorganisms can adhere to surfaces organizing in multicellular communities called biofilms (Costerton et al., 1999). The formation of biofilm is a multi-step process starting with the bacterial attachment to surfaces, and then progressing with the production of extracellular polymeric substances (EPS) (O'Toole et al., 2000; López et al., 2010). The EPS is a complex system constituted of polysaccharides, protein, lipids, nucleic acids and various heteropolymers secreted by the microorganisms in the extracellular environment (Branda et al., 2005; Flemming et al., 2007; Di Martino, 2018).

Within biofilm, bacteria can resist nutrient absence, pH changes better than planktonic organisms (Jefferson, 2004). Moreover, in biofilm state, microorganisms are more resilient to antimicrobial agents compared to planktonic cells (Srey et al., 2013).

Biofilm allows bacteria to bind on a wide range of common food contact surfaces (plastic, stainless steel etc.) and to persist in manufacturing plants after the application of cleaning and disinfection procedures (Hall-Stoodley et al., 2004; Simões et al., 2009). Consequently, once the biofilm is produced, it can be responsible for cross-contamination when food products are exposed to contaminated equipment surfaces (Anand et al., 2014).

Biofilms may develop in a wide range of food processing plants such as meat/poultry (Nikolaev et al., 2022) or dairy (Gopal et al., 2015) and seafood processing (Mizan et al., 2015). Dairy plants could provide an appropriate environment for biofilm development. The temperature, humidity, presence of water, in fact, allows bacteria to grow in dairy processing facilities. In addition, milk waste enriched in fats, proteins, and carbohydrates, could facilitate microbial growth and multiplication (Carrascosa et al., 2021). In addition, it is known that cell hydrophobicity could influence the adhesion of bacteria to a surface and therefore the formation of biofilm (Krasowska and Sigler, 2014).

Under favorable conditions, both spoilage and pathogenic microorganisms may form biofilms and persist in the environment (Galié et al., 2018). As for as the dairy processing plant is concerned, one of the most common biofilm-forming genera is *Bacillus* (Ostrov et al., 2019). *Bacillus* species are ubiquitous, gram-positive, motile, and characterized by a high versatility and adaptability to different environmental conditions (Ehling-Schulz et al., 2015; Gopal et al., 2015). Moreover, they are spore-forming bacteria commonly isolated from both raw and pasteurized milk, pre- and post-pasteurization segments of dairy plants (Shemesh and Ostrov, 2020). Among *Bacillus* species, *B. cereus* may cause food poisoning, because some isolates may produce diarrheal enterotoxins, nonhemolytic enterotoxin (Nhe), cytotoxin K (CytK), hemolysin BL (Hbl), cell wall peptidase (EntFM) and emetic toxin (ces) (StenforsArnesen et al., 2008). It can form biofilm on various materials such as stainless-steel pipes, conveyor belts and storage tanks and it can also form floating or immersed biofilms (Grigore-Gurgu et al., 2019). *B. subtilis*, especially in the biofilm state, can produce a vast array of enzymes, such as heat stable proteases and lipases, which can affect food organoleptic properties of dairy products (Karaka and Güven, 2018). Previous studies investigated biofilm formation of *Bacillus* spp. isolates from dairy sector. To date, few data on molecular characteristics of these biofilm-forming dairy isolates are available (Yuan et al., 2018; Elegbeleye and Buys, 2020; Zhao et al., 2020).

In regard to this, the aims of our study were: i) to evaluate the biofilm forming ability of dairy *Bacillus* spp. isolates (*B. cereus* and *B. subtilis*) in conditions simulating the ones naturally encountered in dairy processing plants; ii) to examine the possible correlation among biofilm formation ability and the hydrophobicity of bacterial cells; iii) to observe the biofilm produced by selected isolates using SEM analysis; iv) to analyze the genetic characteristics of isolates by Whole Genome Sequencing (WGS).

## 2 Materials and methods

### 2.1 Bacterial isolates

*Bacillus* spp. strains (four *B.cereus* and four *B.subtilis*), previously collected from processed cheeses in an Italian dairy processing plant, were used in this study. These isolates were cultured on Plate Count Agar (Oxoid, United Kingdom) by incubation at 30°C and colonies were identified using the Matrix-Assisted Laser Desorption Ionization-Time of Flight (MALDI-TOF) mass spectrometry (Catania et al., 2021). Species’ confirmation was carried out by PCR amplification of the 16S rRNA gene using the universal primers 8F and 533R (Catania et al., 2021). The *B. cereus* ATCC 14579 and *B. subtilis* NCTC 3610 were also included in this study as reference strains.

### 2.2 Screening of biofilm formation of dairy *Bacillus* spp. isolates

The biofilm-forming ability of dairy *Bacillus* spp. isolates and reference strains (*B. cereus* ATCC 14579 and *B. subtilis* NCTC 3610) was evaluated following a previous protocol (Stepanović et al., 2000; Stepanović et al., 2007), with some modifications. All isolates were classified in weak, moderate, strong or no biofilm producers (Stepanović et al., 2000).

In this step, two growth media (Brain Heart Infusion- BHI and Broth and Tryptone Soya Broth - TSB), and two incubation times (24h - 48h), were tested at 30°C to identify the optimal conditions for biofilm formation of the strains included in this experiment.

Briefly, overnight bacterial cultures, in BHI or TSB, were standardized to 0,5 McFarland standard (cell concentration~10^8^ CFU/mL). Subsequently, 200 μL of bacterial suspension was added to 96-well polystyrene microplates (Sarstedt, Germany) with three replicates for each isolate, while the negative control wells contained only the broths. Microtiter plates (96 wells) were incubated for 24 h and 48 h at 30°C. After incubation, medium was discarded and wells were rinsed three times with 300 μL of sterile phosphate buffer saline solution (PBS, pH 7.3, Oxoid, England). Biofilms were heat-fixed at 60°C for 1 h and then stained with 150 μL of 2% w/v crystal violet solution (Chem-lab, Belgium) for 15 min. Unbound crystal violet was discarded by rinsing wells 3 times with distilled water and dried at 37°C for 15 min. To quantify the biofilm formation, 150 μL of 95% ethanol solution (Honeywell, USA) was added to each well. Plates were left at least 30 min at room temperature and then the absorbance was measured at 595 nm on a microplate reader (iMark plate reader, Bio-Rad, Australia). A mean of OD values was calculated for each strain (ODs), while the cut-off OD (ODc) was calculated as three standard deviations above the mean OD of the negative control (Stepanović et al., 2000). According to their OD value, the strains were classified as weak (ODc< ODs ≤ 2 x ODc), moderate (2 x ODc< ODs ≤ 4 x ODc), strong (4 x ODc< ODs) or no (ODs ≤ ODc) biofilm producers. Three independent experiments were performed.

### 2.3 Biofilm formation of dairy *Bacillus* spp. isolates on polystyrene and stainless steel

Dairy *Bacillus* spp. isolates and reference strains, previously classified in weak, moderate or strong biofilm forming strains (see section 2.2), were tested on Nunc™ polystyrene cultures plates (6-well) (ThermoFisher Scientific, USA) (961 mm^2^) and AISI 316 stainless-steel coupons (530 mm^2^) following previous protocols with some modifications (Di Bonaventura et al., 2008; Di Ciccio et al., 2015).

Briefly, overnight bacterial cultures at 30°C in BHI broth (Oxoid, England) were centrifuged at 4000 rpm for 10 min, rinsed thrice with PBS solution (Oxoid, England), and then re-suspended in BHI broth (Oxoid, England). Cultures were diluted to reach 550 nm OD of approximately 0.125 corresponding to a cell concentration of 10^8^ CFU/mL. Three milliliters of each diluted culture were then added to polystyrene tissue culture plates (3 wells for each strain), with or without stainless steel coupons, place horizontally, and then incubated at 30°C for 24 h. After incubation, each PS well was rinsed three times with 3 mL of sterile PBS (Oxoid, England) whereas each SS coupon was rinsed by dipping it three times in different sterile PBS containers to remove non-adherent cells. SS coupons were then, transferred in a new microtiter plate (ThermoFisher Scientific, USA).

After the fixing phase at 60°C for 1 h, three milliliter of crystal violet (2% - Chem-lab Belgium) in 95% ethanol (Honeywell, USA) was added in each well with or without stainless steel coupons. After 20min, the wells were rinsed thrice with distilled water and dried at 37°C for 15 min. Then, 3 ml of a 33% acetic acid solution (Merck, Darmstadt, Germany) were added to each well with or without stainless steel coupons. After 20 min, 0.2 mL from each sample were transferred to a 96-well microtiter plate (Sarstedt, Germany), and the OD level of the destaining solution was measured at 490 nm. Considering the different growth area of tested surface (polystyrene: 961 mm^2^ and stainless steel: 530 mm^2^), results were normalized calculating the biofilm production index (BPI) as follows:


$$BPI=(\frac{ODmean}{biofilm\;surface\;(mm2)})\times 1000$$

#### 2.3.1 Quantification of culturable cells in the total biomass

To determine culturable cells in biofilm, polystyrene wells and stainless-steel coupons were rinsed by dipping 3 times in PBS (Oxoid, England) as previously described (see Section 2.3). Cells were removed using a cell scraper. Serial dilutions were performed in sterile physiological saline peptone (PS) (0.85% NaCl, Carlo Erba; 0.1% Bacteriological Peptone, Oxoid) and spread on BHI-agar plates. Colonies forming units (CFU) were counted after 24 h of incubation at 30°C and the counts were expressed as Log CFU/mm^2^.

### 2.4 Surface hydrophobicity

The hydrophobicity of bacterial cells was evaluated by Microbial Adhesion to hydrocarbon (MATH) assay as previously described by Rosenberg et al., 1980, with some modifications. Briefly, overnight bacterial cultures in BHI were centrifuged and then resuspended in PBS (Oxoid, England) to an optical density of 0.8 at 550 nm, this value represents A1 (Absorbance 1). Followingly, 1 ml of n-hexadecane (Fisher Scientific, Italy) was added to1 ml each bacterial suspension, vortex for 1 minute and incubate at room temperature for 15 min, allowing the separation of aqueous phase. After this time, the absorbance of aqueous phase (Absorbance 2) was measured at 550 nm. Each sample was tested in three independent experiments. The relative hydrophobicity (RH) was expressed as a percentage according to the formula:


$$RH(\%)=(\frac{A1-A2}{A1})\times 100$$

Isolates were classified as: highly hydrophobic for values >50%; moderately hydrophobic for values ranging from 20 to 50% and hydrophilic for values<20%.

### 2.5 Scanning electron microscopy analysis

Biofilm formation was further examined by scanning electron microscopy (SEM). Two reference strains (*B. cereus* ATCC 14579 and *B. subtilis* NCTC 3610) and two dairy isolates (BC_14 and BS_42) were selected for the SEM analysis based on their BPI index.

The microbial cells were grown at 37°C for 24 h on polystyrene tissue plates and stainless-steel coupons (see Section 2.3) then rinsed by dipping 3 times in sterile PBS in order to remove non adherent cells and heat-fixed at 60°C for 1 h. Biomass were fixed with 2.5% glutaraldehyde (Sigma Aldrich, Germany) in 0.1 M sodium cacodylate buffer (pH 7.2) (Sigma Aldrich, Germany) for 30 min at room temperature and then fixed in 1% osmium tetroxide (Sigma Aldrich, Germany) for 1 h. Samples were then rinsed with 0.1 M cacodylate buffer for 1 h to remove any unreacted glutaraldehyde before rinsing and dehydration. Samples were dehydrated through an ascending series of ethanol (30%, 50%, 70% and 80%) for 15 min for each concentration, and then three times for 15 min in 100% ethanol. Finally, they were overnight air-dried. Specimens were then sputter-coated with a gold-palladium layer using an Emitech K575X Peltier-cooled (EM Technologies, England). Finally, selected samples were observed using the Jeol LV300 Scanning Electron Microscope at an accelerating voltage of 25kV and working distance of 6 mm, with a probe current of about 100 pA. All SEM analyses were performed in two independent experiments.

### 2.6 Extraction of genomic DNA and whole genome sequencing of *Bacillus* isolates

For DNA extraction, isolated colonies were inoculated in brain heart infusion (BHI) broth and incubated at 30°C, overnight. The DNA of *B. cereus* and *B. subtilis* strains was extracted using the DNeasy Blood and Tissue Kit (Qiagen Ltd., UK), according to the manufacturer’s instructions. DNA concentration and purity were determined respectively using the Qubit Fluorometer (Thermo Fisher Scientific, USA) with the Qubit dsDNA HS Assay Kit and NanoDrop spectrophotometer (ThermoFisher Scientific, USA).

Library preparation, WGS and control steps were performed by genomics service company Novogene (Cambridge, United Kingdom). Samples were run on Illumina NovaSeq 6000 platform to generate 2 × 150 bp paired end (PE) reads.

Reads were first trimmed for low quality (Phred score< 20) by using SolexaQA++ software v3.1.7.1 (Cox et al., 2010), and reads shorter than 60 bp were discarded with PRINSEQ v0.20.4 (Schmieder and Edwards, 2011). Clean reads were then assembled using SPAdes v3.14.1 (Nurk et al., 2017) and contigs shorter than 500 nt were discarded. Genes were annotated with Prokka v1.14.5 (Seemann, 2014) with default parameters. QUAST v5.0.2 software was then used to check the quality of the contigs (Gurevich et al., 2013).

Draft genomes of *B. cereus* ATCC 14579 and *B. subtilis* NCTC 3610 were downloaded from NCBI, and genes were annotated with Prokka. The pan genome calculation and phylogenetic analysis of *Bacillus* strains were obtained by Roary v. 3.11.2 (Page et al., 2015). Phylogenetic tree was then visualized in iTOL6 (https://itol.embl.de/).

Venn diagrams were obtained by Venn Diagram Maker (https://goodcalculators.com/venn-diagram-maker/).

Predicted genes were then aligned against genes involved on biofilm formations by BLASTn tools (e-value cutoff of 1e-5, requiring a hit to display >90% of identity over at least 30% of the query length).

### 2.7 Statistical analysis

The data shown are average values obtained in at least three independent experiments with standard deviations. For assessment of significant differences, one-way analysis of variance (ANOVA) with Tukey’s *post hoc* test was performed. Statistical analyses and graphing were conducted with GraphPad Prism version 8.4.3 (GraphPad Software, San Diego,CA, USA). Differences were considered statistically significant when *p* values were less than 0.05.

## 3 Results

### 3.1 Screening of biofilm formation of dairy *Bacillus* spp. isolates

The biofilm formation of dairy isolates (four *B. cereus* and four *B. subtilis*) and reference strains (*B. cereus* ATCC 14579 and *B. subtilis* NCTC 3610) was evaluated in BHI broth (Oxoid, England) and TSB (Oxoid, England) for 24h and 48h, at 30°C.

**Figure 1:**
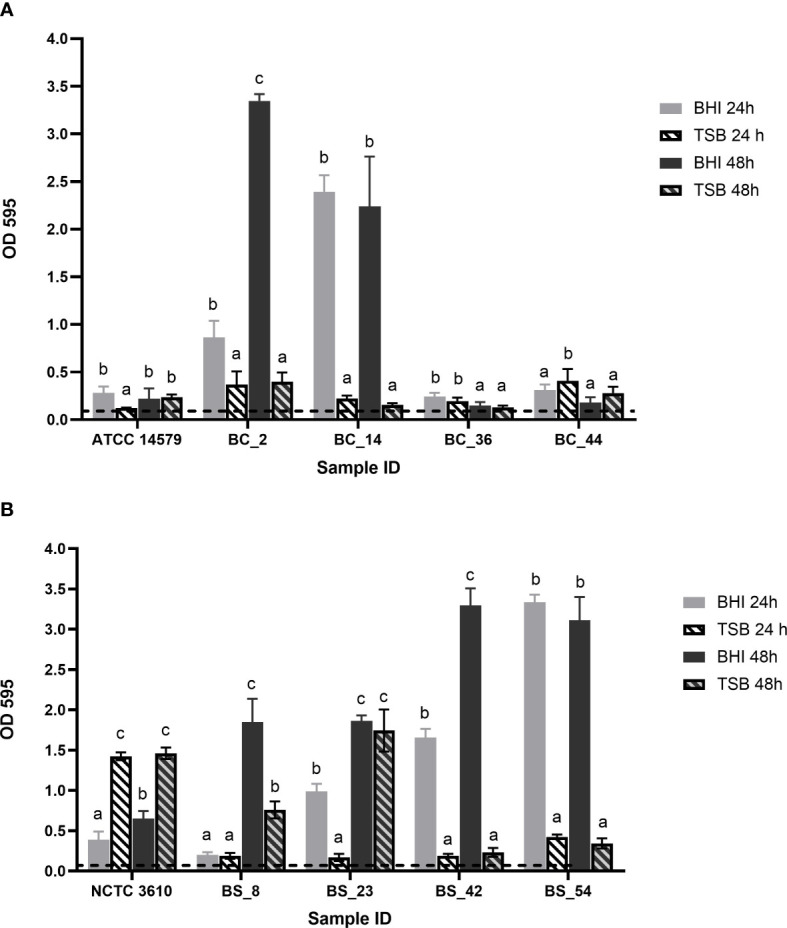
Biofilm forming ability of **(A)** *Bacillus cereus* (BC) dairy isolates and ATCC 14579 reference strain and **(B)** *Bacillus subtilis* (BS) dairy isolates and NCTC 3610 reference strain in different growth media (BHI and TSB) and incubation time (24h and 48h). The threshold of biofilm formation (dotted line) is equal to OD values ≤ 0.11. Error bars indicate standard deviation. Different letters indicated significant difference among the isolates, p< 0.05.

After 24h, all isolates were able to produce biofilm, with statistically significant differences (*p*< 0.05) in relation to the growth media and incubation time (**Figure 1**).

According to their OD values (see Section 2.2),*B. cereus* BC_2 and BC_14 isolates were classified as strong biofilm producers in BHI, at 24 and 48h, whereas they are respectively moderate and weak producers in TSB, with no statistically significant differences as a function of the incubation time (**Figure 1A**). Strain BC_36 was a weak producer in all conditions; BC_44 isolate was classified as a moderate biofilm producer, with a biofilm production slightly higher in TSB after 24h, but without statistical difference compared to the same time in BHI (**Figure 1A**). ATCC 14579 reference strain was a moderate producer in BHI after 24h, whereas at the same incubation time, a weak biofilm production was assessed in TSB, no statistically significant difference was highlighted for the two growth media after 48h (**Figure 1A**).

On the other hand, all *B. subtilis* isolates resulted strong producers in BHI, except BS_8 strain which after 24h in BHI, resulted as weak biofilm producer (**Figure 1B**). The reference strain NCTC 3610showed moderate biofilm-forming ability in BHI, while in TSB was classified as strong producer (**Figure 1B**).

### 3.2 Biofilm formation of dairy *Bacillus* spp. isolates on polystyrene and stainless steel

**Figure 2:**
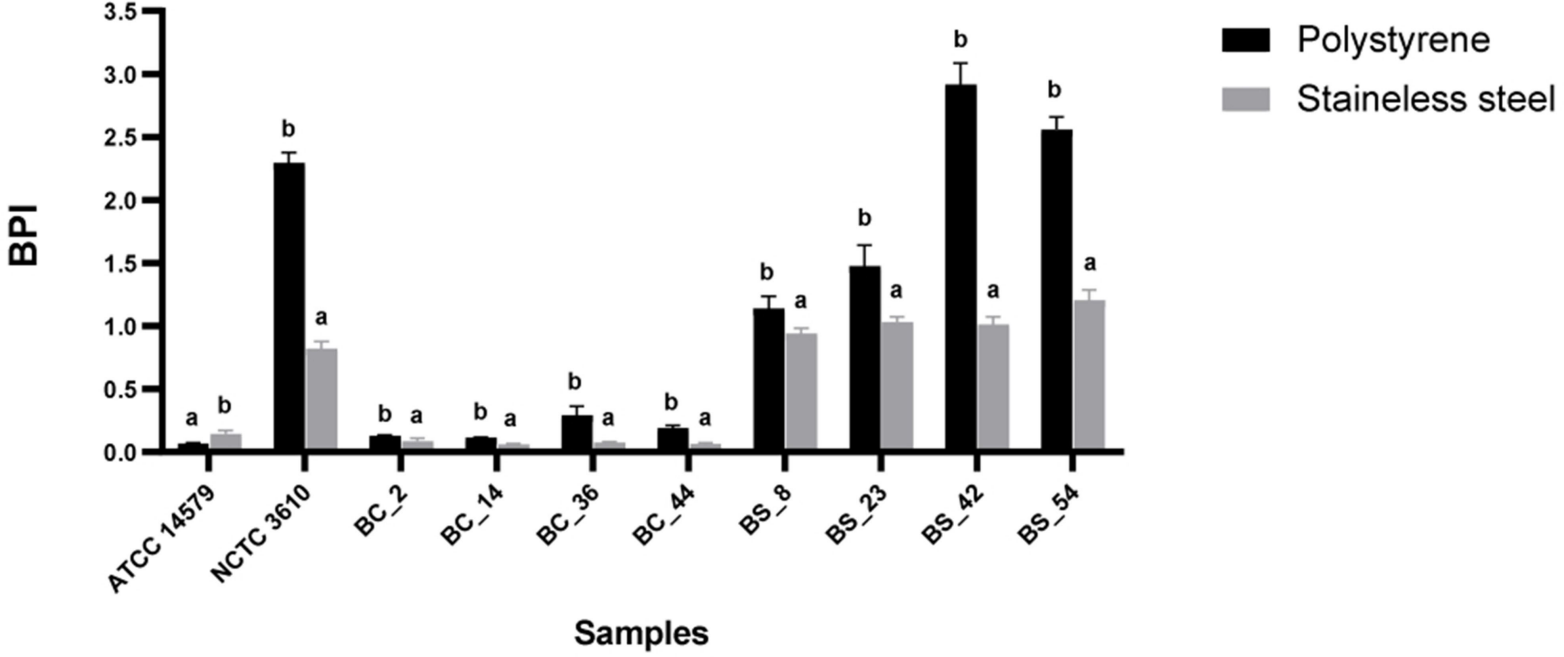
Biofilm formation of reference strains ATCC 14579 and NCTC 3610, *Bacillus cereus* (BC) and *Bacillus subtilis* (BS) food isolates, on polystyrene and stainless-steel coupons. Biofilms were formed in BHI at 30°C for 24 h and biofilm production index (BPI) was measured by the crystal violet assay. Each bar represents the result of the average of three biological experiments. Error bars indicate standard deviation. Letters on the top of the bars indicate significant statistically differences (*p*< 0.05).

The BPI index of each strain was evaluated on two common food contact surfaces: polystyrene and stainless steel at 30°C(see Section 2.3). Reference strains (*B. cereus*ATCC 14579 and *B. subtilis*NCTC 3610) were included. A higher biofilm production on polystyrene was observed for all isolates, except for *B. cereus* ATCC 14579 (**Figure 2**). The BPI indices of *B. subtilis* were higher than *B. cereus* in both examined surfaces (**Figure 2**).

### 3.3 Quantification of culturable Bacteria in the total biomass

**Figure 3:**
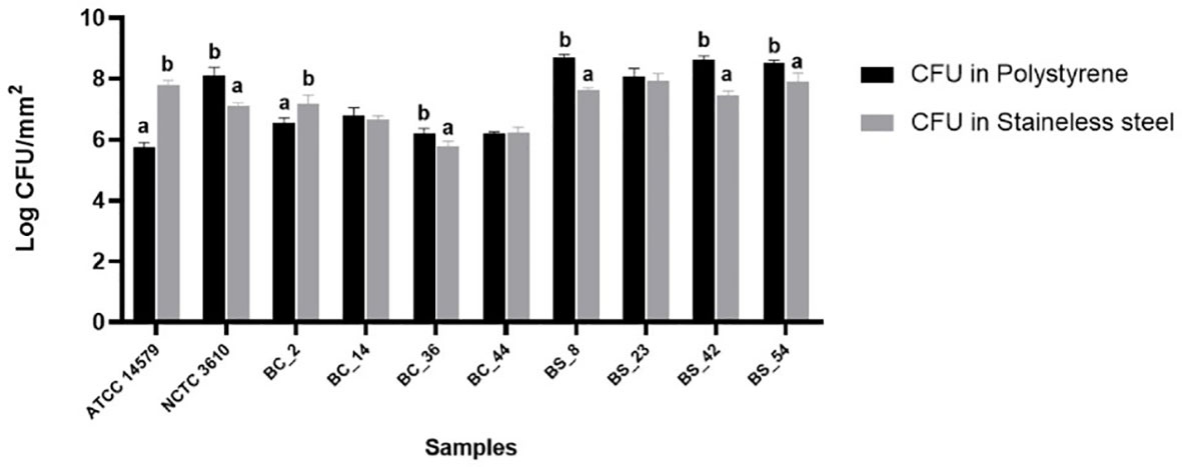
Total CFU counts in the biofilms of reference strains, *B. cereus* (BC) and *B. subtilis* (BS). Biofilms were grown on polystyrene wells and stainless-steel coupons in BHI at 30°C for 24 h. Each bar represents the result of the average of three biological experiments and standard deviation for each strain. To compare the number of sessile cells yielded among strains, one-way ANOVA and Tukey’s *post hoc* test were performed. Groups with different alphabets indicate significant difference (*p<* 0.05).

Cell counts were performed to assess the number of culturable cells in the biomass of each sample. The load of *Bacillus cereus* cells ranging from 5,74 ± 0,16 and 6,78 ± 0,27 Log CFU/mm^2^ on polystyrene wells and 5,77 ± 0,18 and 7,81 ± 0,13 Log CFU/mm^2^ on stainless steel coupons respectively. Among materials (polystyrene and stainless steel) used in this study, a statistically significant difference was observed, except for BC_44 isolate (**Figure 3**). The reference strain ATCC 14579 showed the lowest bacterial count on polystyrene and highest bacterial count on stainless steel (**Figure 3**), in accordance with BPI values (see Section 3.2).

As for as *Bacillus subtilis* is concerned, cell counts ranged from 8,08 ± 0,26 to 8,7 ± 0,10 Log CFU/mm^2^ on polystyrene wells, and from 7,12 ± 0,08 and 7,95 ± 0,23 on stainless steel coupons. A statically significant difference was observed among materials, except for BS_23 isolate (**Figure 3**).

### 3.4 Surface hydrophobicity

With MATH assay, the cell surface hydrophobicity of dairy isolates and reference strains was tested. The percentage of hydrophobicity, jointly to the BPI values in polystyrene and stainless steel, is reported in **Table 1**. Among the tested strains, all *B. subtilis*, including NCTC 3610 reference strain, were evaluated as hydrophobic. A variability in the percentage of adhesion was observed for *B. cereus* strains: two isolates (BC_36 and BC_44) revealed a hydrophobic character, one strain resulted hydrophilic (BC_14), and other two (BC_2 and *B. cereus* ATCC 14579 reference strain) moderately hydrophobic (**Table 1**).

**Table 1:** Hydrophobicity of *B. cereus* and *B. subtilis* including the reference strains (ATCC 14579 and NCTC 3610).

Sample ID	Species	% Hydrophobicity	BPI polystyrene	BPI stainlesssteel
ATCC 14579	*Bacillus* *cereus*	36,3 ± 0,08	0,07 ± 0,01	0,15 ± 0,03
BC 2		39,7 ± 0,02	0,13 ± 0,01	0,08 ± 0,02
BC 14		14,0 ± 0,09	0,11 ± 0,00	0,06 ± 0,00
BC 36		81,3 ± 0,01	0,29 ± 0,07	0,07 ± 0,01
BC 44		79,0 ± 0,04	0,19 ± 0,03	0,07 ± 0,01
NCTC 3610	*Bacillus* *subtilis*	82,9 ± 0,10	2,29 ± 0,08	0,82 ± 0,06
BS 8		85,0 ± 0,08	1,14 ± 0,10	0,94 ± 0,04
BS 23		85,0 ± 0,12	1,47 ± 0,17	1,03 ± 0,04
BS 42		83,0 ± 0,11	2,92 ± 0,17	1,01 ± 0,06
BS 54		83,0 ± 0,07	2,56 ± 0,10	1,2 ± 0,09

Hydrophobicity was determined using hexadecane. The percentage of hydrophobicity of the strains was correlated to the biofilm production index (BPI) in polystyrene and stainless steel. Values reported represent the average of three experiments ± standard deviation.

### 3.5 Scanning electron microscopy analysis

To observe biofilm architecture of *Bacillus* spp., SEM analysis was performed. Two isolates of dairy origin (*B. subtilis*BS_42 and *B. cereus*BC_14) were selected based on their BPI index on polystyrene and stainless steel. Reference strains were also included in this analysis.

**Figure 4:**
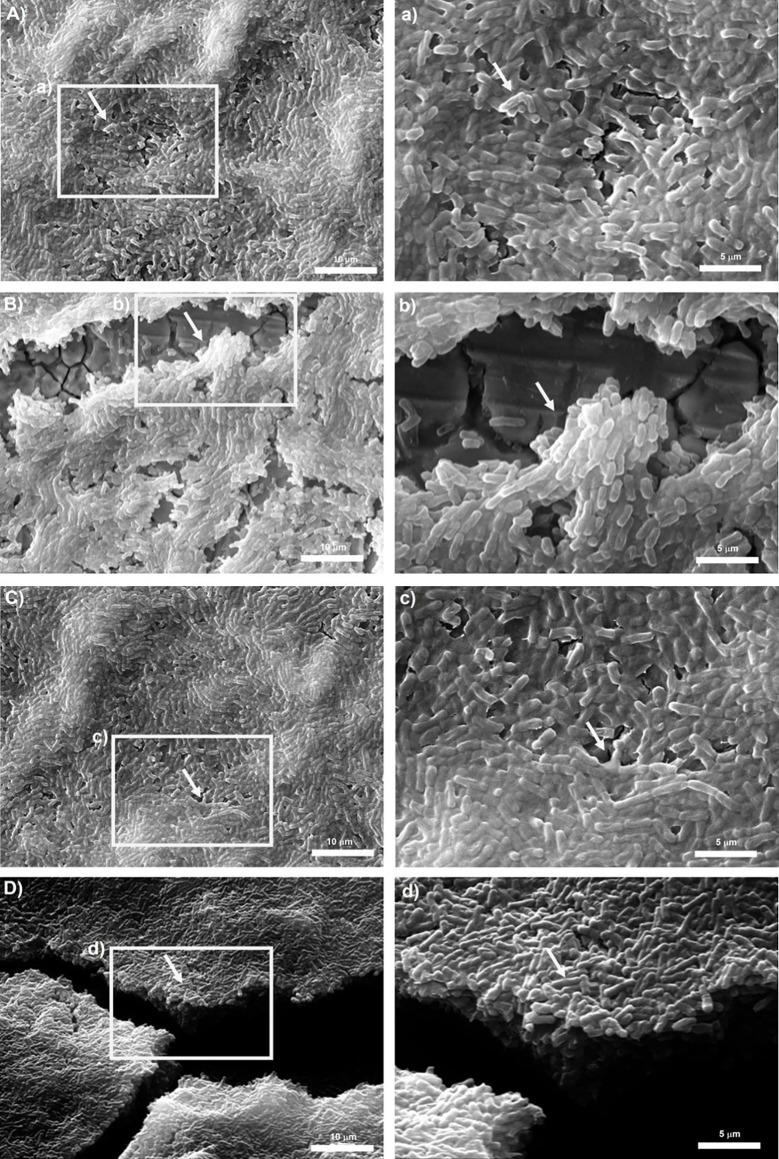
Scanning electron microscope (SEM) images of biofilms formed by reference *B. subtilis* strain NCTC 3610 in **(A)** polystyrene and **(B)** stainless-steel and dairy *B. subtilis* isolate BS_42 in **(C)** polystyrene and **(D)** stainless-steel. On the left the representation of 2000x, on the right 5000x magnification. The white arrows show the EPS matrix. Biofilms were formed in BHI broth at 30°C for 24h. a,b,c,d: structure details and biofilm matrix visualized by SEM.

**Figure 5:**
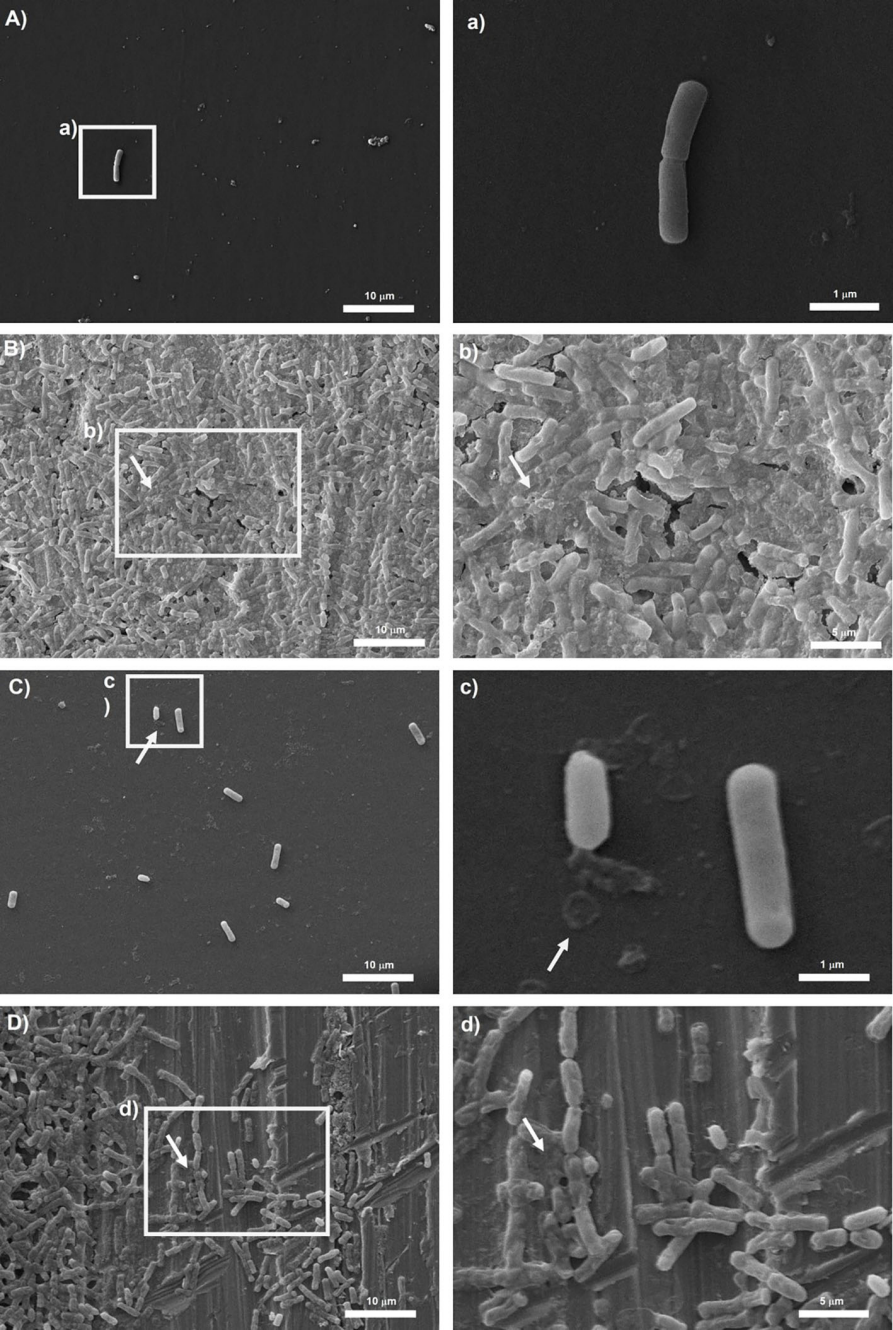
Scanning electron microscope (SEM) images of biofilms formed by reference *B. cereus* strain ATCC 14579 in **(A)** polystyrene and **(B)** stainless-steel and dairy *B. cereus* isolate BC_14 in **(C)** polystyrene and **(D)** stainless-steel. On the left the representation of 2000x, on the right 10000x magnification for (a, c) and 5000x magnification for (b, d). The white arrows show the EPS matrix. Biofilms were formed in BHI broth at 30°C for 24h.

Representative micrographs of biofilms produced by tested isolates are shown in **Figures 4**, **5**. *Bacillus subtilis* reference strain NCTC 3610 and dairy isolate BS_42, showed a complex tridimensional meshwork-like structure of cells at high density, embedded in a network of extracellular polymeric substances on polystyrene surface compared to stainless steel surface (**Figure 4**). Considering *B. cereus*, fibrillar materials were visible on polystyrene for reference strain ATCC 14579 and dairy strain BC_14, (**Figures 5A, C**), whereas a partial biofilm consisting of sparse aggregates of cells bound by few or absent extracellular polymeric substances was observed on stainless steel (**Figures 5B, D**).

### 3.6 Genetic characterization of isolates based on WGS

Strains were *de novo* sequenced, assembled and subjected to comparative analysis. *B. cereus* genomes display a GC content between 35,09 and 35,16% and a length ranging from 5.7 Mb to 6.4 Mb (**Table 2**). *B. subtilis* genomes display a GC content between 38.74 and 43.66% and a length ranging from 4.1 Mb to 9.5 Mb (**Table 2**).

**Table 2:** Whole genome sequencing data of dairy *B. cereus* and *B. subtilis* tested isolates.

Sample ID	Species	ro	Number of contigs	Largestcontig	Total length (bp)	N50 (bp)	N75 (bp)	GC (%)	CDS	rRNA	tRNA	tmRNA
BC_2	*B. cereus*	3,48	82	667936	5798536	228431	127999	35.16	5712	4	62	1
BC_14		3,54	94	721757	5971488	243905	84197	35.12	5836	3	74	1
BC_36		3,59	99	721755	6055942	244060	78184	35.11	5832	4	70	1
BC_44		3,23	198	621663	6402612	191779	78593	35.09	5976	4	74	1
BS_8	*B. subtilis*	3,44	338	721721	9571502	78184	29454	38.74	9632	4	86	2
BS_23		3,35	82	265522	4027612	95564	43403	43.68	4114	3	65	1
BS_42		4,95	83	265522	4073463	95564	46046	43.66	4183	3	71	1
BS_54		4,04	81	265522	4119257	99153	46046	43.66	4181	3	71	1

**Figure 6:**
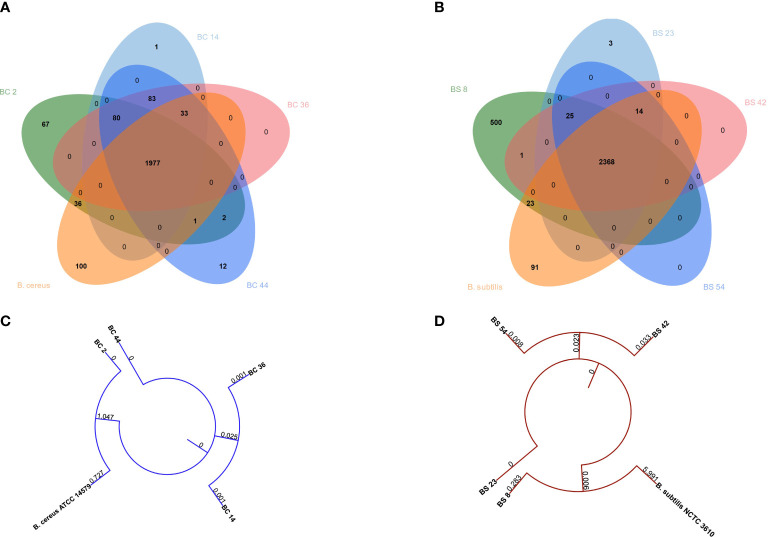
Venn diagrams depicting the overlap between genes common or unique between **(A)** *B. cereus* or **(B)** *B. subtilis* genomes. Phylogenetic tree built on concatenated **(C)** *B. cereus* or **(D)** *B. subtilis* genes.

A potential functional diversity was observed across the different strains. Venn diagram depicted the overlap among *B. cereus* genes common or unique between strains (**Figure 1A**). 1977 genes were shared among strains. BC_14 isolate displayed the unique presence of *Cytochrome f* gene, BC_44 the presence of 12 unique genes, BC_2 of 67 genes, while type strain showed the presence of 100 unique genes (**Figure 6A**)

When comparing *B. subtilis* strains, a total of 2368 genes were shared, while three were unique to BS_23, 500 to BS_8 and 91 were unique in the type strain (**Figure 6B**).

Phylogenetic tree showed that *B. cereus* genomes BC_36 and BC_14 and BC_2 and *B. cereus* ATCC 14579 clustered together, while BC_44 belonged to a different cluster (**Figure 6C**). The same behavior was observed between *B*. *subtilis* genomes BS_42 and BS_54 and BS_8 and *B. subtilis* NCTC3610, while BS_23 genome was different (**Figure 6D**).

The presence of biofilm related genes in tested isolates was evaluated extrapolating the data from WGS (**Table 3**); moreover, for *B. cereus* strains, toxin profile was also assessed (**Table 4**).

**Table 3:** Biofilm related genes of *B. cereus* and *B. subtilis* isolates included in the study.

Genes	*Bacillus cereus*					*Bacillus subtilis*					Description	References
	BC 2	BC 14	BC 36	BC 44	ATCC 14579	BS 8	BS 23	BS 42	BS 54	NCTC 3610		
*spo0A*	–	–	–	–	+	+	+	+	+	+	Sporulationtranscriptionfactor Spo0A	Chastanet and Losick, 2011
*comER*	–	–	–	–	+	–	+	+	+	+	Putative pyrroline-5’-carboxylate reductase	Yan et al. (2016)
*codY*	+	–	–	–	+	+	+	+	+	+	Transcriptional regulator, GTP and BCAA-dependent	Lindback et al. (2012)
*plcR*	–	+	+	+	+	+	–	–	–	–	Phospholipase C accessory protein PlcR	Hsueh et al. (2006)
*abrB*	+	+	+	+	+	+	+	+	+	+	Transcriptional regulator for transition state genes	Fagerlund et al. (2014)
*calY*	–	+	+	+	+	+	–	–	–	–	Biofilm matrixprotein	Caro-Astorga et al. (2014)
*sipW*	–	–	–	–	+	+	+	+	+	+	Type I signalpeptidase	
*epsG*	–	–	–	–	–	+	+	+	+	+	Transmembrane proteinEpsG	Branda et al., 2001
*epsH*	–	–	–	–	–	+	+	+	+	+	Putative glycosyltransferaseEpsH	

Results +/- indicate presence or absence of genes examined.

**Table 4:** Toxin profile of *B. cereus* isolates included in the study.

Genes	*Bacillus cereus*				Description	References
	BC 2	BC 14	BC 36	BC 44		
*hbl*	–	–	–	–	hemolyticcomplex BL (HBL)	Owusu-Kwarteng et al. (2017)
*nhe*	+	+	+	+	non-hemolysinenterotoxincomplex	Guinebretière et al. (2010)
*entFM*	+	+	+	+	enterotoxin FM	Ngamwongsatit et al. (2008)
*bceT*	–	–	–	–	enterotoxin T	Guinebretière et al. (2002)
*cytK*	+	+	+	+	cytotoxin K	Ngamwongsatit et al. (2008)
*ces*	–	–	–	–	cerulide	Ehling-Schulz et al. (2006)

Results +/- indicate presence or absence of genes examined.

Food isolates display a variety of biofilm-regulated genes. The frequency of these genes was higher in *B. subtilis* compared to *B. cereus*, with differences depending on strains (**Table 3**).

The presence of hemolysin *hbl* was not detected, all isolates were positive for non-hemolysin enterotoxin *nhe*. Positivity for *entFM* and *cytK* was also detected in all analyzed bacterial isolates. In contrast, the *bceT* gene and emetic strains (*ces* gene) were not identified (**Table 4**).

## 4 Discussion

Although the presence of *Bacillus* spp. biofilms on equipment surfaces in dairy processing industries have been reported by several authors, few studies described the differences among *Bacillus* spp. isolates on the ability to form biofilms (Hayrapetyan et al., 2015; Elegbeleye and Buys, 2020). Members of the *Bacillus* genus are considered one of the most harmful dairy biofilm formers, which may threaten the quality and safety of milk products (Shemesh and Ostrov, 2020), thanks to their ability to resist to severe thermal processing (Hanson et al., 2005; Luu et al., 2015).

In previous research, we demonstrated the presence of *Bacillus* isolates in processed cheese, hence survived to a series of heat treatment (Catania et al., 2021). In this research, we evaluated their ability to produce biofilm.

It is well known that the formation of biofilm can be influenced by various factors, including time of incubation and composition of growth media (Wijman et al., 2007). To determine the optimal conditions for biofilm production, we evaluated the ability of these dairy *Bacillus* spp. isolates and reference strains to form biofilm in different environmental conditions, i.e., different growth media (BHI and TSB) and time of incubation (24h - 48h) at the optimal temperature (30°C) by using a previously described biofilm method (Stepanović et al., 2000; Stepanović et al., 2007). This practice represents one of the most common approaches used as screening method to assess biofilm formation ability of bacterial isolates (Azeredo et al., 2017). Our results showed that after 24h all tested strains were able to produce biofilm, with differences depending on growth media and incubation time (see section 3.1). A slight amount of biofilm formation was assessed after 24h in BHI for ATCC 14579 reference strain. *B. cereus* BC_2 and BC_14 dairy isolates were more effective in biofilm formation in BHI broth, compared to TSB, while BC_36 strains was a weak producer in both growth media and, for BC_44 isolate, no statistically significant difference was observed after 24 h in BHI broth and TSB. After 24 h, all *B. subtilis* isolates were strong biofilm produced in BHI, compared to TSB, except *B. subtilis* BS_8 strain and NCTC 3610 reference strain. Our results are in accordance with previous studies in which the biofilm production of food isolated strains was higher in BHI (Hayrapetyan et al., 2015; Kowalska et al., 2020). Conversely, in another study authors showed that there was no difference in biofilm formation in BHI and TSB for *B. cereus* strains after 24 h, whereas after 48 h of incubation, the biofilms formed were significantly higher in TSB than BHI (Kwon et al., 2017). A possible explanation of a greater biofilm production in BHI, assessed for the most of tested isolates, could be related to its richness of nutrients. The high number of proteins contained in BHI, especially leucine, proline, serine, and aspartate, as well as the presence of lipids such as choline, sphingosine and sugars, may increase the biofilm expression (Stepanović et al., 2000). In addition, since after 24h in BHI most of tested isolates were classified as strong/moderate biofilm producers, for the next experiments (macro-method assay), we evaluated the biofilm production after 24h of incubation.

Various studies reported the ability of bacteria to adhere on different surfaces, including polystyrene (PS), stainless steel (SS), glass, rubber, aluminum, titanium, and ceramic (Donlan, 2001; Simões et al., 2010). We investigated the biofilm formation of *Bacillus* spp. isolates on PS and SS. The choice of polystyrene was based on its extensive use in the food industry mainly for packaging purposes (Genualdi et al., 2014), whereas stainless steel is widely employed in food industry equipment, such as tanks and pipes (Dewangan et al., 2015). To simulate conditions the naturally encountered in industrial plants, the experiments carried out after the screening method were performed by using 6-well plates. This system overcomes the limitation of the basic microtiter plate assay (96 wells format) concerning possible nutrient limitation and the inability to observe biofilm structure by direct microscopy (SEM). In addition, it increases the surface area for biofilm formation. In accordance with the literature, our results showed that the surface properties affected the biofilm production by *Bacillus* spp. isolates (Simões et al., 2010). Biofilm formation was higher on PS compared to SS for all tested isolates, except for *B. cereus* ATCC 14579. Previous studies reported a robust biofilm formed by ATCC 14579 on SS, compared with plastic and glass surface (Kwon et al., 2017). The great biofilm formation was particularly evident for *B. subtilis* strains (see Section 3.2). On the contrary, previous studies on *Bacillus* spp. of dairy origin reported a higher biofilm production on SS compared to PS (Hayrapetyan et al., 2015; Kwon et al., 2017); whereas in a recent study authors highlighted the ability of dairy-related *B. subtilis* isolates to form moderate and/or strong biofilms on PS (Elegbeleye and Buys, 2020).

To evaluate a possible correlation among the biomass, expressed as BPI index, and the number of viable cells, total bacterial counts (CFU) were performed both in PS and SS. Our results showed that there was not a correlation among CFUs and BPI indices. These findings are in accordance with other studies which highlighted that biofilm biomass is not directly related to viable bacterial cells (Monteiro et al., 2015; Stiefel et al., 2016; Rodríguez-Lázaro et al., 2018; Panebianco et al., 2021). This result could be explained since the crystal violet staining, used to measure the total biomass, includes both viable and non-viable bacteria, as well as the polymeric matrix (Monteiro et al., 2015; Stiefel et al., 2016; Rodríguez-Lázaro et al., 2018; Panebianco et al., 2021).

The variation in biofilm production on PS and SS could be related to the physicochemical characteristics of the substrates which could influence microorganisms’ adhesion (Legeay et al., 2010). Previous studies highlighted that biofilm formation on hydrophobic substrata (PS) occurred greater than that on hydrophilic ones (SS) (Cerca et al., 2005; Pagedar et al., 2010; Di Ciccio et al., 2015). The hydrophobicity seems to be a relevant factor contributing to the biofilm formation ability, and it plays a role in the early stage of bacterial adhesion, for this reason we investigated the relationships among bacterial cell hydrophobicity and biofilm formation of dairy isolates. All tested *B. subtilis* isolates, including NCTC 3610 reference strain, were highly hydrophobic (see **Table 1**). These data agreed with the high BPI values observed on PS (see **Figure 2** and **Table 1**). Interestingly, *B. cereus* strains showed greater variability i.e., two isolates were classified as hydrophobic, two as moderately hydrophobic and one as hydrophilic (see **Table 1**). In a recent study, Elegbeleye and Buys (2020) observed that bacterial cell surface hydrophobicity is strain-specific, and intrinsic and/or extrinsic factors play a role in the biomass and biofilm’s structure. Overall, *B. subiltis* isolates were the species more adherent to hexadecane, and they developed more abundant biofilms on the more hydrophobic biomaterial (polystyrene). On the contrary, less abundant biofilms were observed for the most hydrophilic strains. In this sense, hydrophobicity seems influence biofilm formation.

Development of biofilm is closely associated with the generation of EPS, mainly composed of polysaccharide material. Several microscopy techniques have been developed aimed at a deeper understanding about the composition, properties, and function of biofilm formation (Wolf et al., 2002; Neu and Lawrence, 2014). Among them, SEM microscopy has been employed by several authors to observe the structure of biofilm-forming bacteria (El Abed et al., 2012). Thus, in our study to observe the presence of EPS in *Bacillus* spp. strains organized in biofilm state, we performed a SEM microscopic analysis of a 24-h-old mature biofilm of selected isolates based on their BPI values. Briefly, we attempted to correlate the biofilm formation, expressed as BPI indices, with the SEM images. The SEM analysis revealed a considerable amount of exopolysaccharide matrix on both surfaces in *B. subtilis* isolate and reference strain (NCTC 3610) as shown in **Figure 4**. This observation was in accordance with the BPI values calculated for these strains. On the contrary, the biofilm architecture and structure formed by *B. cereus* reference strain (ATCC 14579) and a dairy selected isolate was less complex on polystyrene (see **Figure 5**) compared to stainless steel (24 h of incubation at 30°C) in terms of amount of extracellular polysaccharides produced. These findings were evident by SEM analysis especially for ATCC 14579 (see **Figure 5**) and in accordance with BPI values obtained.

Genetic profile of isolates was also assessed by WGS analysis. Results showed a similarity of strains among the same species, both for *B. cereus* and *B. subtilis*. Considering toxin profile in food isolates, all *B. cereus* strains carrying *nhe* genes that are associated with diarrheal syndrome (Guinebretière et al., 2010), previous studies reported that *B. cereus* strains isolated from food surfaces harbored *nhe* genes (Carter et al., 2018). Moreover, *entFM* and *cytK* genes were also identified, a similar result was reported by Tirloni for *cytK* gene (Tirloni et al., 2020). Conversely, *hbl*, *bceT* and *ces* genes were not detected. The presence of emetic strains in the dairy production chain is considered rare (Svensson et al., 2006). Anyway, the positivity for toxin genes is not necessarily correlated to their production, and thus, further studies should be performed to elucidate this issue.

Moreover, the presence of selected genes related to biofilm formation was investigated and a significant difference among the two species was assessed. Only *abrB* gene, known to play a central role in biofilm formation by regulating cell mobility and differentiation, was identified in all tested isolates (Ma et al., 2017). *Spo0A* and *comER* genes were not detected in *B. cereus* strains while they were present in all *B. subtilis*, except for one dairy isolate where *comER* was absent. The *comER* gene plays an important role in the regulation of biofilm formation and sporulation in both *B. subtilis* and *B. cereus* (Yan et al., 2016). Results of a recent study carried on both bacterial species, suggest that *comER* may be part of the regulatory pathway that controls activation of *Spo0A* (Yan et al., 2016). *Spo0A* governs the genetic pathway controlling the matrix production, its importance as key regulator of biofilm formation was described in *B. subtilis* and *B. cereus* (Gao et al., 2015). In the study of Gao et al. (2015) produced a mutant with a deletion of *Spo0A*, they examined the ability of this mutant strain to generate spore and form biofilm by microscopy analysis and we reported that Δspo0A strain exhibited a defect in sporulation and biofilm production. Regarding *comER*, in 2016 Yan and colleagues (2016) investigated the role of this gene, showing that *comER* mutant in *B. cereus*, as much as in *B. subtilis* strains, showed a defect in biofilm formation. The *sipW* gene encodes a peptidase that is specifically required for the processing and secretion of the encoded EPS matrix proteins (Branda et al., 2006). Our results showed its presence only in *B. subtilis* strains. Furthermore, in all *B. cereus*, except dairy isolate BC_2, the *codY* gene was not identified, whereas it is present in all *B. subtilis*. In a previous study, authors showed that in *B. cereus* strains which lack *codY* gene, a decreased biofilm forming capacity was shown (Hsueh et al., 2008). On the other hand, the *plcR* and *calY* genes were mainly identified in *B. cereus* isolates. The development of biofilm is associated with EPS production (Branda et al., 2001). Among genes involved in matrix production, *epsG* and *epsH* were also examined and only observed in *B. subtilis* isolates. The biofilm matrix of *B. cereus* is similar to other *Bacillus*, but unlike *B. subtilis*, genes responsible for the EPS synthesis are not essential for *B. cereus* (Gao et al., 2015). Molecular analysis showed a greater presence of genes related to biofilm formation for *B. subtilis* strains, this result agreed with phenotypic results. However, despite the presence of biofilm-related genes in the genome of isolates, the concrete expression level of these genes remains unknown. Hence, additional gene expression studies are necessary to elucidate this issue.

## 5 Conclusions

The management of microbial contamination in the context of the dairy processing industry is crucial. *Bacillus* spp. such as *B. subtilis* and *B. cereus* can survive even after severe heat treatments, and they are known to be biofilm forming bacteria in the dairy equipment. The presence of *Bacillus* spp. biofilm is considered a significant concern to the dairy industry because it represents a potential source of continuous contamination in the working environment with implications for the safety and spoilage of dairy products. In the current study, all *B. subtilis* and *B. cereus* strains, isolated from processed cheese products and surviving after the heat treatments, were able to form biofilm on common food contact surfaces, with species-specific variation. *B. subtilis* strains produced a robust biofilm compared to *B. cereus* isolates. These differences can be correlated with presence or absence of biofilm related determinants in the genome, and they are also influenced by cell surface properties. Moreover, the SEM analysis highlighted a complex biofilm structural architecture and an extracellular matrix which covered and embedded the bacterial cells. It is known that *Bacillus* spp. strains organized in a biofilm state are more difficult to eradicate than planktonic cells. Regarding this, biofilm produced by *Bacillus* spp. in pipeline and tank systems in dairy processing equipment represents a big issue because of their resistance to common cleaning in place practices routinely applied in many dairy plants. Considering these aspects, the importance of expanding our knowledge on biofilm formation and its relationship with the molecular characteristics of isolates is crucial to better understand the complex mechanisms underlying this phenomenon. Finally, further investigations should be performed to elucidate molecular mechanisms involved in biofilm formation of *Bacillus* spp. to find novel strategies to minimize the risk of biofilm in dairy processing equipment.

## Data availability statement

The datasets presented in this study can be found in online repositories. The names of the repository/repositories and accession number(s) can be found below: https://www.ncbi.nlm.nih.gov/, PRJNA90254.

## Author contributions

AC: investigation, methodology, data curation, formal analysis, writing—original draft. AD and PC: conceptualization, supervision, writing—review and editing. IF: data curation, bioinformatics analysis, review and editing. TC: resources and funding acquisition and project administration. FC: methodology, review and editing. All authors contributed to the article and approved the submitted version.
